# Combining Image Analysis, Genome Wide Association Studies and Different Field Trials to Reveal Stable Genetic Regions Related to Panicle Architecture and the Number of Spikelets per Panicle in Rice

**DOI:** 10.3389/fpls.2016.01384

**Published:** 2016-09-20

**Authors:** Maria C. Rebolledo, Alexandra L. Peña, Jorge Duitama, Daniel F. Cruz, Michael Dingkuhn, Cecile Grenier, Joe Tohme

**Affiliations:** ^1^Agrobiodiversity, International Center for Tropical AgriculturePalmira, Colombia; ^2^Agricultural Research for Development - CIRAD, Unités Mixtes de Recherche - Amélioration Génétique et Adaptation des PlantesMontpellier, France

**Keywords:** GWAS, inflorescence architecture, yield, rice, direct seeding, image analysis

## Abstract

Number of spikelets per panicle (NSP) is a key trait to increase yield potential in rice (*O. sativa*). The architecture of the rice inflorescence which is mainly determined by the length and number of primary (PBL and PBN) and secondary (SBL and SBN) branches can influence NSP. Although several genes controlling panicle architecture and NSP in rice have been identified, there is little evidence of (i) the genetic control of panicle architecture and NSP in different environments and (ii) the presence of stable genetic associations with panicle architecture across environments. This study combines image phenotyping of 225 accessions belonging to a genetic diversity array of *indica* rice grown under irrigated field condition in two different environments and Genome Wide Association Studies (GWAS) based on the genotyping of the diversity panel, providing 83,374 SNPs. Accessions sown under direct seeding in one environement had reduced Panicle Length (PL), NSP, PBN, PBL, SBN, and SBL compared to those established under transplanting in the second environment. Across environments, NSP was significantly and positively correlated with PBN, SBN and PBL. However, the length of branches (PBL and SBL) was not significantly correlated with variables related to number of branches (PBN and SBN), suggesting independent genetic control. Twenty- three GWAS sites were detected with *P* ≤ 1.0E-04 and 27 GWAS sites with *p* ≤ 5.9E−04. We found 17 GWAS sites related to NSP, 10 for PBN and 11 for SBN, 7 for PBL and 11 for SBL. This study revealed new regions related to NSP, but only three associations were related to both branching number (PBN and SBN) and NSP. Two GWAS sites associated with SBL and SBN were stable across contrasting environments and were not related to genes previously reported. The new regions reported in this study can help improving NSP in rice for both direct seeded and transplanted conditions. The integrated approach of high-throughput phenotyping, multi-environment field trials and GWAS has the potential to dissect complex traits, such as NSP, into less complex traits and to match single nucleotide polymorphisms with relevant function under different environments, offering a potential use for molecular breeding.

## Introduction

Growing world population and the effects of global climate change are increasing the demand for higher crop yields. To meet rice demand in 2050, annual increase production has to increase from the current 1–2.4% by 2050 (Ray et al., 2013). New rice varieties with high yield potential have been a major target of crop improvement in rice (Peng et al., 1999). A global tendency has been to shift from puddled-transplanted rice production to direct seeding (DRS) of rice in irrigated systems to save labor and water resources (Kumar and Ladha, 2011). In Asia and Africa farmers are considering shifting to DRS (Farooq et al., 2011), however varieties currently used for DRS were bred for transplanted culture in puddle soils.

Under both production systems increasing sink size at flowering is a priority trait to increase yield potential in rice (Dingkuhn et al., 1991, 2015; Foulkes et al., 2011). One approach to increase sink size at flowering is to increase the number of spikletes per panicle (Peng et al., 1999). The panicle consists of a main axis called rachis with primary, secondary, and higher order branches bearing the spikelets. The number and dimensions of branches vary among cultivars (Ikeda et al., 2004), and will define the final architecture of the panicle. High number of secondary and primary branches and long primary branches were associated with high number of spikelets per panicle in rice (Miura et al., 2010; Fujita et al., 2013). Therefore, the genetic improvement of the number of spikelets per panicle may benefit from its dissection into component traits of lesser genetic complexity, such as panicle architecture traits (AL-Tam et al., 2013; Crowell et al., 2014).

The genetic control of panicle architecture plays an important role in rice production. Large variation was observed for panicle architecture traits among five different rice sub species (Crowell et al., 2014, 2016). To date, several genes and QTLs related to panicle architecture and affecting the number of spikelets per panicle (as reviewed by Sreenivasulu and Schnurbusch, 2012; Liang et al., 2014) have been reported. Genes such as *Gn1a, OsSPS1*, and *SPIKE* with a positive effect on the number of secondary branches also showed a significant effect on the number of spikelets per panicle and yield (Ashikari et al., 2005; Fujita et al., 2013; Hashida et al., 2013). Many QTLs related to the number and length of secondary branches with small but cumulative phenotypic effect on the number of spikelets per panicle were reported (Ando et al., 2008; Peng et al., 2014). In addition, the length of primary branches has been shown to be positively related to the number of spikelets per panicle (Li et al., 2009). Understanding the relationship between panicle architecture traits and the number of spikelets per panicle will help to identify trait combinations related to a high number of spikletes per panicle that could be used for breeding.

A panicle is a complex structure and the characterization of the branching pattern for breeding has also been limited by the unavailability of appropriate screening tools. Recently, open access image analysis software packages were released to analyze panicle architecture (AL-Tam et al., 2013; Crowell et al., 2014), allowing a rapid description of each panicle architecture trait. The study of large diversity panels using Genome Wide Association Studies (GWAS) allows capturing the available allelic diversity on the loci of interest with a high physical map resolution due to the small linkage disequilibrium (LD). It is also an efficient way to dissect the genetic architecture of complex traits and a powerful tool for crop breeding (Norton et al., 2014; Rebolledo et al., 2015; Ueda et al., 2015). Using both methods (GWAS and image analysis), Crowell et al. (2016) revealed a large number of GWAS sites controlling panicle architecture. However, the relation with the final number of spikelets per panicle was not established, and relevant traits such as secondary branch number and length (Xu et al., 2004) were not addressed.

Identifying the genes responsible for intraspecific variation in the number of spikelets per panicle has been challenging because of the polygenic nature and the environmental sensitivity of panicle architecture traits. In fact, inconsistent detection and variable effects of QTLs controlling the number of spikelets per panicle across environments in rice (Zhuang et al., 1997; Li et al., 2012) revealed genotype by environment (GXE) interactions for this complex trait. Panicle architecture traits are also stongly influenced by management practices (Huang et al., 2011; Dixit et al., 2015), temperature (Laza et al., 2015), and drought (Pantuwan et al., 2002). Thus, the detection of QTLs depends on both the genetic background (parental combinations) and the environment where the experiments were conducted, resulting sometimes in partly non-overlapping sets of QTLs (Li et al., 2012; Wade et al., 2015). These results point to the necessity of assessing the genetic and phenotypic control of the number of spikelets per panicle and panicle branching for specific genetic backgrounds and environments. To our knowledge this has not been done for panicle architecture and the number of spikelets per panicle in rice.

Rice breeding will need tools to determine the ideal panicle architecture to increase the number of spikelets per panicle either under a specific or for a broad range of conditions. This study aims at extracting functional concepts to understand the physiological and genetic control of panicle architecture and the number of spikelets per panicle using an indica diversity panel, an image phenotyping tool and two contrasting environments to reveal GWAS sites that are either environement specific or stable across the environments.

## Materials and methods

### Association mapping panel

The population studied represented the diversity within the indica sub-species (http://ricephenonetwork.irri.org) covering improved and traditional varieties from all tropical regions. For the present study, the association mapping panel consists of a sub-sample of 225 indica accessions obtained from the International Rice Research Institute (IRRI) seedbank. Among the 225 accessions, 51 originate from Africa, 12 from South and Central America, 120 from South Asia and 42 from West and East Asia (Table S1).

### Phenotypic evaluation

#### Site description and crop establishment

The experiments were conducted under irrigated conditions during the dry season (May to August) in CIAT Palmira (76° 21′W, 3° 30′N, and 967 m elevation). The experiment was established under transplanting conditions (Exp1 TR) in 2013 and under direct seeding (Exp2 DRS) in 2014. For both experiments, the soil was a silt-loam soil with particle distribution of 10% clay, 80% silt, and 10% sand. The experimental design was a randomized complete block design with 3 replications and 225 plots. The 225 accessions were sown at the same time and grouped by previously estimated maturity date and plant height to facilitate measurements.

In 2013, seeds from the 225 accessions were sown in plastic trays and seedlings transplanted, 20 days after emergence, at one seedling per hill with a hill spacing of 0.2 × 0.2 m. Plot size was 3.0 × 0.6 m, for a plot density of 25 plants/m^2^. Plots were maintained under lowland conditions with a water depth of 5 cm, after transplanting until 2 weeks after flowering. Phosphorus and potassium were applied at 59.8 kg ha^−1^ P_2_O_5_ and 116.6 kg ha^−1^ K_2_O. A total of 207.4 kg ha^−1^ N, 20.8 kg ha^−1^ Fe, and 2.8 kg ha^−1^ Zn was applied. The top soil contained 27.5 g organic C kg^−1^ and 1.3 total N kg^−1^ with pH 8.2.

In 2014, mechanical sowing was done in dry soil with a plot size of 3.0 × 1.0 m and a plot density of 80 plants/m^2^. Sprinkler irrigation was applied after sowing. Fertilizers used were 145.9 kg ha^−1^ of N, 40.5 kg ha^−1^ P_2_O_5_, 17.6 kg ha^−1^ Fe, and 2.8 kg ha^−1^ Zn. Twenty days after sowing the field was maintained under lowland condition with a water depth of 5 cm, until 2 weeks after flowering. The top soil contained 20.5 g organic C kg^−1^ and 1.18 total N kg^−1^ with a pH 7.5.

For both experiments weeds were controlled manually, and by herbicide applications at pre and post-emergence.

Weather data was collected daily, at one-meter distance from the experimental trial, and with a 30 min time step in a data logger connected with radiation (silicon pyranometer sensor (S-LIB-M003), temperature and humidity sensors (S-THB-M002) (Onset Computer Corporation, Bourne, USA). Average solar radiation, minimum and maximum temperature, minimum and maximum humidity for each experiment are presented in supplementary table (Table S2).

#### Measurements

##### Panicle architecture

In both experiments, the main stem of two plants in each plot was tagged 30 days after sowing. At flowering tagged stems were harvested with its respective panicles. Each panicle was spread out on a white background and held in place with metal pins under a sticky paper. A total of 1350 panicles were dissected using P-TRAP software (AL-Tam et al., 2013). Panicle length (PL), number of primary (PBN) and secondary (SBN) branches, and length of primary (PBL) and secondary branches (SBL) were extracted from image analysis (Table 1, Table S3).

**Table 1:** **Trait analyzed on the diversity panel of Indica rice**.

**Variable**	**Name**	**Measurement unit**
PBN	Primary branch number	Number/panicle
SBN	Secondary branch number	Number/panicle
PBL	Primary branch length	cm/panicle
SBL	Secondary branch length	cm/panicle
PL	Panicle length	cm/panicle
NSP	Number of spikelets per panicle	Number/panicle

##### Number of spikelets per panicle

At harvest, panicles were sampled from central rows, within a soil base area of 0.2 m^2^ in Exp1-TR and of 0.125 m^2^ in Exp2-DRS. Filled and unfilled spikelets per panicle were separated from the rachis manually, and counted using the seed counter (Data Count S JR, Data Technologies) (Table S3).

#### Statistical analysis

The descriptive analysis and analysis of variance were performed with SAS v9.3 (SAS Institute, Cary NC, USA) with PROC MEAN and PROC MIXED, respectively. ANOVA test was applied considering genotype as fixed effect and block and group as random effect. Means were adjusted for blocks and group factor. Histograms of frequency distribution were conducted with PROC SGPLOT and broad-sense heritability was computed using PROC MIXED model in SAS. Pearson correlation was performed using R v3.1.2 (www.R-project.org).

### Genotypic data

The panel included in this study is a subset of 329 indica accessions that were genotyped using the genotype by sequencing (GBS) protocol (Elshire et al., 2011) at Cornell University. Raw reads were demultiplexed and aligned to the rice reference genome (Os-Nipponbare-Reference-IRGSP-1.0) (Kawahara et al., 2013), and variants were identified using the NGSEP pipeline (Duitama et al., 2014). This procedure provided a raw catalog of 690 thousand variable sites across the genome.

From these panel, the following filters were applied to build a curated SNP dataset available for GWAS of 91,591 SNPs with 22.8% of missing data: minor allele frequency (MAF) ≥ 0.05 and observed heterozygosity ≤ 0.01 (Perea et al., 2016). Missing data was imputed with the implementation of the FastPhase Hidden Markov Model (Scheet and Stephens, 2006) available at NGSEP. Comparison with SNP calls from whole genome sequencing (WGS) data from 27 varieties included in the 3000 rice genomes project (3KRGP, The 3000 rice genomes project, 2014) allowed to predict an estimated error rate of 0.85% for the non-imputed dataset and of 1.2% for the imputed dataset. Finally, genotype data for the 225 accessions phenotyped in this study was selected from the imputed dataset and a MAF filter (MAF > 0.05) was executed again to avoid spurious associations due to low frequency alleles, leading to a final dataset of 83,374 SNPs.

### Genomic wide association analysis

Association mapping was performed using TASSEL v5.2.8, a genotypic matrix of 83,374 SNPs and a phenotypic matrix with 225 accessions. To detect associations between SNP and panicle architecture related traits a mixed linear model (MLM) was applied involving a kinship matrix as a random effect generated using the pairwise identity by state distance implemented in TASSEL v5.2.8.

Plots representing GWAS results (Manhattan and Quantile-Quantile plots) were performed using the packages QQman in R 3.2.2 (www.R-project.org).

Significance threshold for association detection was set to *P* < 1.1E−04. Within a 75 kb region (LD reported in the *indica* sub-specie, Mather et al., 2007) any SNPs and genes linked to the detected peak with a strong LD (*r*^2^ > 0.7) were considered as a unique region and defined as a GWAS site. To identify genes potentially linked to the detected SNPs, we used the gene database Gramene, a comparative resource for plants (www.gramene.org).

## Results

### Range of variation in the diversity panel for the number of spikelets per panicle and panicle architecture under contrasting conditions

We observed significant differences (*p* < 0.001) for all measured variables between both experiments (Table 2). Average NSP was 143 in Exp1-TR and 102 in Exp2-DRS and average PL was 23.98 cm in Exp1-TR and 22.06 cm in Exp2-DRS (Table 2). For panicle architecture related traits, PBN, SBN and SBL showed higher values in Exp1-TR than in Exp2-DRS while PBL had higher values in Exp2-DRS than in Exp1-TR.

**Table 2:** **Means and broad sense heritability for each trait measured in both experiments**.

	**Exp1-TR**			**Exp2-DRS**		
**Trait**	**Mean**	**s.d**	**H2**	**Mean**	**s.d**	**H2**
PBN	15.55a	2.25	0.87	10.16b	1.41	0.81
SBN	37.73a	11.17	0.81	25.33b	7.58	0.79
PBL	9.27b	1.24	0.87	10.28a	1.4	0.84
SBL	2.63a	0.32	0.9	2.48b	0.31	0.89
PL	23.98a	3.03	0.84	22.06b	2.67	0.76
NSP	143.11a	34.32	0.81	102.57b	22.71	0.77

*Tukey test was used to calculate differences between years*.

*Means with different letters are significantly different at p < 0.0001*.

**Figure 1:**
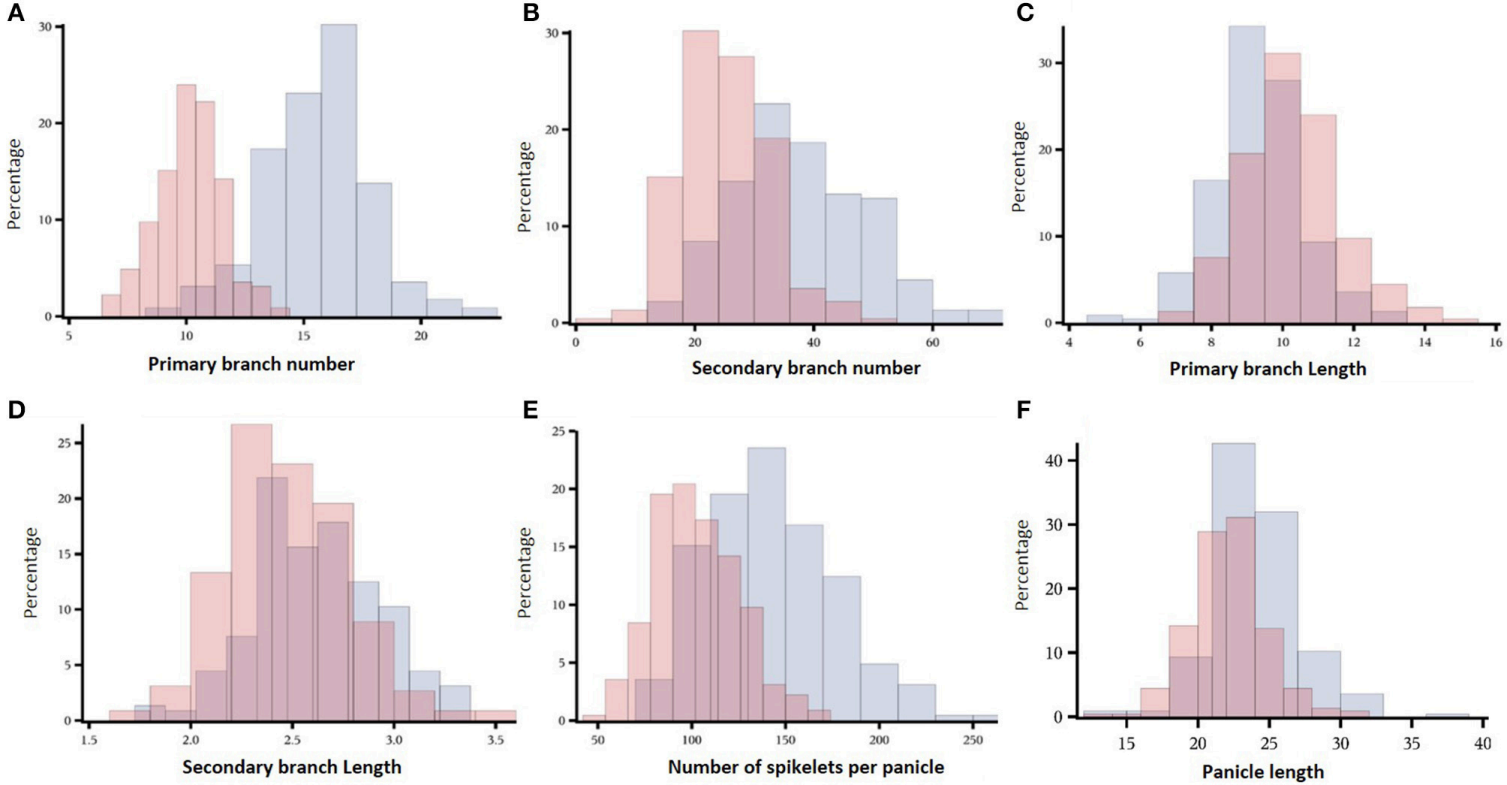
**Histograms of the distribution for panicle architecture related traits in both experiments**. In blue and red are values for Exp1-TR and Exp2-DRS respectively. The percentage of individuals for each class is presented in the y-axis. **(A)** Primary branch number, **(B)** Secondary branch number, **(C)** Primary branch length, **(D)** Secondary branch length, **(E)** Number of spikelets per panicle, **(F)** Panicle length.

Image analysis allowed to identify panicle architecture differences within the *indica* diversity panel (Figure 1). The genotypic variation for NSP was large, ranging from 51 to 169 spikelets per panicle in Exp2-DRS and from 73 to 258 spikelets per panicle in Exp1-TR (Figure 1E). PL differences between varieties ranged from 13.3 to 37.9 cm in Exp1-TR and from 13.1 to 31.5 cm in Exp2-DRS. PBN values ranged from 9 to 22 in Exp1-TR and from 6 to 14 in Exp2-DRS (Figure 1A). PBL, SBL, and SBN showed a wide distribution for each experiment (Figures 1B–D), however differences between experiments were lower than those observed for PBN. Since the experiments were conducted under different sowing conditions, we computed broad sense heritability for each experiment. All the measured variables showed broad sense heritability values higher than 0.7 (Table 2).

### Correlations between panicle architecture traits and the number of spikelets per panicle

For all variables, the correlation between Exp1-TR and Exp2-DRS across genotypes was positive and significant (*p* < 0.001) (Table 3). The highest correlation between experiments was observed for NSP and SBL while the lowest correlation was observed for PBL and PL (Table 3).

**Table 3:** **Correlation between panicle architecture and number of spikelets per panicle for both experiments**.

	**NSPexp1**	**NSPexp2**	**PBLexp1**	**PBLexp2**	**PBNexp1**	**PBNexp2**	**SBLexp1**	**SBLexp2**	**SBNexp1**	**SBNexp2**	**PLexp1**	**PLexp2**
NSPexp1	1											
NSPexp2	0.682^**^	1										
PBLexp1	0.184^*^	0.126	1									
PBLexp2	0.138	0.167^*^	0.473^**^	1								
PBNexp1	0.545^**^	0.527^**^	0.058	0.119	1							
PBNexp2	0.489^**^	0.491^**^	−0.124	−0.099	0.582^**^	1						
SBLexp1	0.112	0.002	0.672^**^	0.403^**^	−0.098	−0.352^**^	1					
SBLexp2	0.079	0.035	0.406^**^	0.770^**^	−0.015	−0.244^*^	0.619^**^	1				
SBNexp1	0.598^**^	0.559^*^	0.365^**^	0.118	0.719^**^	0.383^**^	0.145	0.002	1			
SBNexp2	0.589^**^	0.607^*^	−0.029	0.354^**^	0.483^**^	0.541^**^	−0.101	0.107	0.580^**^	1		
PLexp1	0.094	0.099	0.635^**^	0.435^**^	0.338^**^	−0.044	0.421^***^	0.307^***^	0.398^***^	0.095	1	
PLexp2	0.141	0.180^*^	0.427^**^	0.538^**^	0.074	0.276^**^	0.226*	0.349^**^	0.161	0.350^**^	0.400^**^	1

Values with ^***^, ^**^, and ^*^ indicate correlations with p < 0.0001, p < 0.01, and p < 0.05, respectively. Bold values indicate significant correlations between traits.

Correlations between variables showed similar patterns for Exp1-TR and Exp2-DRS (Table 3): (i) PL was significantly and positively correlated to all the measured panicle architecture traits (PBL, PBN, SBL, SBN) (Table 3), (ii) PBN was positively and significantly (*p* < 0.001) correlated with SBN, (iii) PBL was positively and significantly (*p* < 0.001) correlated with SBL, and (iv) PBL was significantly (*p* < 0.001) and positively correlated with SBN.

Interestingly, PBN was not significantly correlated with PBL, and SBN was not significantly correlated with SBL in any experiment. Suggesting that, considering the same rank of branching, greater branch number was not associated with greater branch length.

Secondary branches originate from primary branches therefore it was not surprising that PBL was significantly and positively correlated with SBN (Table 3).

NSP was positive and significantly (*p* < 0.01) related to both the number and length of branches (PBL, PBN, and SBN) within both Exp1-TR and Exp2-DRS; but no significant correlation was observed between NSP and SBL (Table 3). The correlation between NSP and variables related to the number of branches (PBN,SBN) was higher than the correlation with variables related to the length of branches (PBL) (Table 3). PL was not significantly correlated with NSP in Exp1-TR, while the correlation between PL and NSP in Exp2-DRS was positive and significant (*p* < 0.05). Since NSP was not significantly correlated with PL, PL was not considered for the GWAS study.

### GWAS for panicle architecture traits and the number of spikelets per panicle

In the final dataset of 83,374 SNPs the number of SNPs available for analysis resulted in a mean distance between neighboring SNPs was 4.7 kbp. The coverage of the indica genome could thus be considered as saturating when considering a linkage disequilibrium spanning 75 kbp on average in this population (Mather et al., 2007). The genome wide map of SNP variation thus gave a good coverage of the genome.

**Figure 2:**
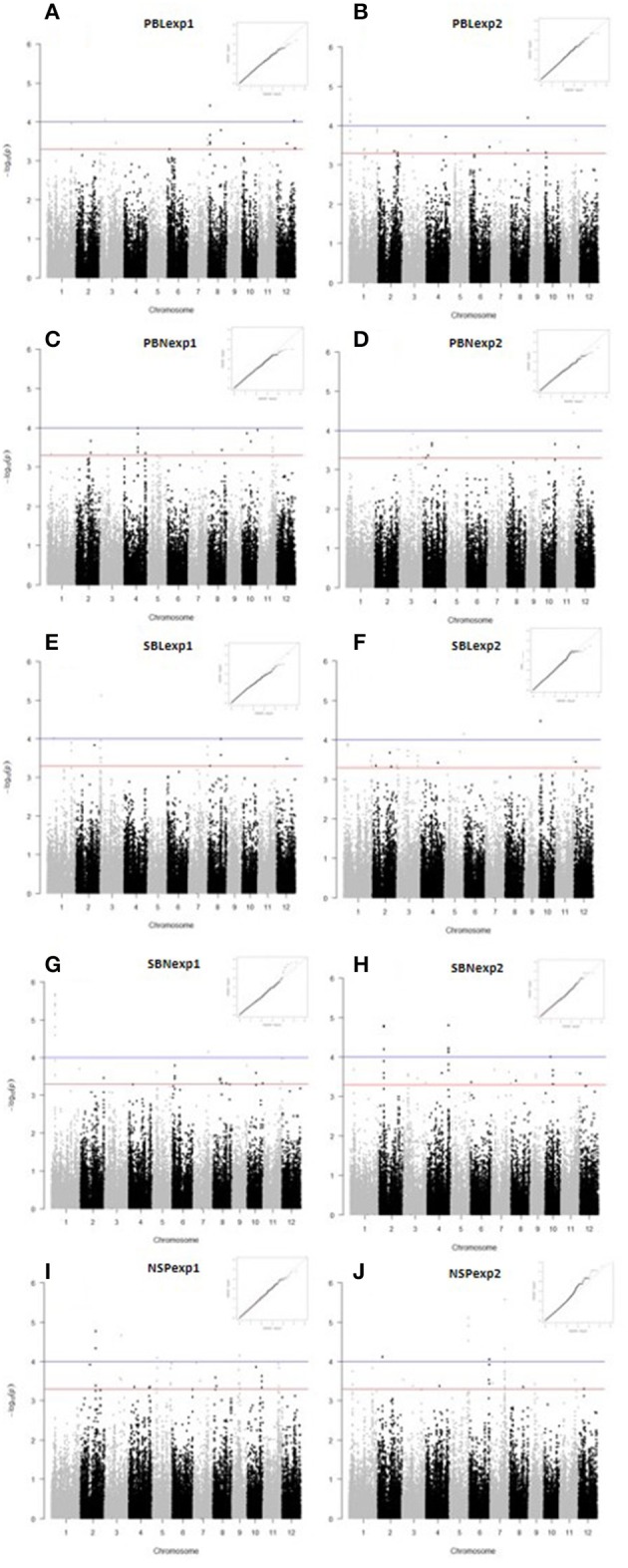
**Manhattan plots and Q-Q plots for panicle architecture related traits and number of spikelets per panicle. (A)** PBLexp1, **(B)** PBLexp2, **(C)** PBNexp1, **(D)** PBNexp2, **(E)** SBLexp1, **(F)** SBLexp2, **(G)** SBNexp1, **(H)** SBNexp2, **(I)** NSPexp1, and **(J)** NSPexp2. In the Manhattan plots the blue line and red lines indicates the threshold for significant SNP association at *p* < 1 × 10^−4^ and *p* < 5 × 10^−4^ respectively.

**Figure 3:**
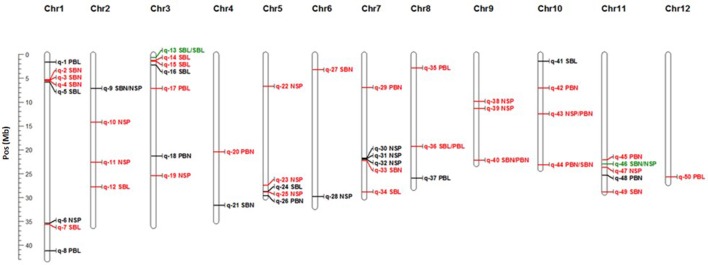
**Physical map position of significant GWAS sites detected through MLM1**. In red GWAS sites for Exp1-TR, in black GWAS site for Exp2-DRS and in green GWAS sites across experiments. Co-locations are represented by “/” between traits.

We used a compressed MLM to identify association signals. The Manhattan plots and Quantile-Quantile plots of all the traits are shown in Figure 2. Twenty three GWAS sites were significantly associated with panicle architecture and NSP with a threshold of *p* < 1.0E−04, and 27 with *P* = 5.9E−04 (Figure 3, Table S4).

A total of 14 GWAS sites were associated with NSP, 17 GWAS sites were associated with branch length (6 to PBL and 11 to SBL), and 19 GWAS sites were associated with the number of branches (11 with SBN, and 8 with PBN). NSP collocated with panicle architecture variables only in the cases of SBN in Exp2-DRS (q-9) and PBN in Exp1-TR (q-43) (Figure 3). No collocation was observed between NSP and PBL or SBL within the same experiment. Finally, no GWAS collocations occurred between the number of branches (SBN, PBN) and the length of branches (SBL, PBL) (Figure 3).

### Trait, markers and known genes associations

A total of 21 GWAS sites were located near known genes that have been previously identified using mutants or studies of recombinant populations (Table 4).

**Table 4:** **List of known cloned genes related with panicle traits located within the linkage dis equilibrium block of significant GWAS sites detected in this study**.

**GWAS site**	**Traits**	**Chr**	**Locus name**	**Gene name**	**Pos gene (MSU)**	**Function**	**References**
q-1	PBLexp2	1	LOC_Os01g03840	*Osjag*	1625159–1626771	Floral organ development.	Duan et al., 2010
q-2	SBNexp1	1	LOC_Os01g10040	*d2*	5236623–5244011	Brassinosteroid biosynthesis. Leaf angle. Grain size.	Hong et al., 2003
q-3	SBNexp1	1	LOC_Os01g10110	*OsCKX2*	5270103–5275678	Grain number. Cytokinin catabolism.	Ashikari et al., 2005
q-6	NSPexp2	1	LOC_Os01g61044	*OsAAT7*	35320780–35323282	Carbon and nitrogen content.	Lu et al., 2012
q-7	SBLexp1	1	LOC_Os01g61480	*lax*	35558148–35559225	Lateral organ development. Axillary meristem formation.	Komatsu M. et al., 2003
q-12	SBLexp1	2	LOC_Os02g45770	*Osmads6-5*	27875979–27884079	Floral organ identity. Formation of the incipient primordia of lodicule, stamen and carpel.	Duan et al., 2012
q-14	SBLexp1	3	LOC_Os03g03070	*OSMADS50*	1269856–1271783	Flowering time.	Lee et al., 2004
q-15	SBL exp1	3	LOC_Os03g03150	*te*	1327397–1331210	Tillering. Dwarfism. Twisted flag leaf.	Lin et al., 2012
q-16	SBLexp2	3	LOC_Os03g04680	*OsCYP96B4*	2223102–2225205	Dwarfism. Cell elongation. Pollen germination.	Ramamoorthy et al., 2011
q-17	PBLexp1	3	LOC_Os03g13010	*TUD1*	7029149–7031702	Dwarfism. Grain size. Leaf morphology.	Hu et al., 2013
q-20	PBNexp1	4	LOC_Os04g33740	*GIF1*	20422171–20427062	Grain filling. Grain size.	Wang et al., 2008
q-22	NSPexp1	5	LOC_Os05g11730	*GSK2*	6657481–6661493	Dwarfism. Leaf size. Tillering. Brassinosteroid signaling. Delayed flowering. Dense panicle. Leaf angle. Seed size.	Tong et al., 2012
q-23	NSPexp1	5	LOC_Os05g47780	*OsHRZ2*	27377537–27382230	Fe acquisition.	Kobayashi et al., 2013
q-23	NSPexp1	5	LOC_Os05g47840	*OsIPT7*	27422988–27424795	Tiller growth. Root development. Cytokinin biosynthesis.	Sakamoto et al., 2006
q-25	NSPexp1	5	LOC_Os05g50270	*nl1*	28817605–28818871	Floral organ identity. Flowering time. Growth retardation. Culm starch content. Germ cell development. Meiosis.	Wang et al., 2009
q-26	PBNexp2	5	LOC_Os05g51670	*OsUGE1*	29631973–29635357	Carbon partitioning during nitrogen limitation.	Guevara et al., 2014
q-27	SBNexp1	6	LOC_Os06g06750	*OsMADS5*	3162801–3169415	Floral organ formation.	Cui et al., 2010
q-28	NSPexp2	6	LOC_Os06g49250	*OsPTR9*	29838283–29841400	Ammonium uptake. Lateral root formation. Grain size. Panicle size.	Fang et al., 2013
q-36	PBLexp1, SBLexp1	8	LOC_Os08g31219	*S27-1*	19304739–19306021	Hybrid sterility between Oryza sativa and Oryza glaberrima. Pollen development. Interaction with S28.	Yamagata et al., 2010
q-37	PBLexp2	8	LOC_Os08g40930	*OsISA1*	25892391–25900576	Seed starch content.	Utsumi et al., 2011
q-44	PBNexp1, SBNexp1	10	LOC_Os10g42690	*jmj6*	23025714–23031771	Number and morphology of floral organ.	Sun and Zhou, 2008

Six GWAS sites associated with panicle architecture and the number of spikelets per panicle were located within the LD distance of 6 genes related to floral organ formation (Table 4). *NECKLEAF1 (nl1)* a gene controlling floral organ identity and flowering time was located within the LD block of q-25 associated with NSPexp1. *Osmads6-5* and *Lax* genes affecting the number of spikelets per panicle and the number of branches were within the LD block of q-12 and q-7 respectively and associated with SBLexp1. *Osmads5* was within the LD region of q-27 associated to SBNexp1, *OsJAG* gene which has a known function on the development of the panicle and the number of spikelets per panicle was near q-1 in chromosome 1 position 1.626.152 bp and associated with PBL in Exp2. Finally, *Jmj6* controlling the number and morphology of reproductive organs was within the LD region of q-44 associated with PBNexp1. One GWAS site q-14 was near a gene controlling flowering time, SBLexp1 in chromosome 3 was within the LD block of the gene *OSMADS50*.

Three GWAS sites q-6, q-23, q-26, q-28 associated with the number of spikelets per panicle or the number of primary branches were within the region of genes controlling plant nutrient status (ammonium, Iron, nitrogen content). Genes controlling plant size were within the LD region of GWAS sites associated with NSP or with SBL (q-22, q-15, q16, q23). The sites q-2, q-3, q-17, q-20, q-36, q-37 were within the LD regions of genes controlling grain size, pollen development or seed starch content suggesting a linkage between the genetic control of panicle architecture and grain formation.

Therefore, for all the panicle traits dissected with image analysis (PBL, PBN, SBL, and SBN), we found significant associations at sites where known genes with relevant function were reported.

### GWAS sites detected across experiments

Thirty one GWAS sites were associated with variables measured in Exp1-TR and 17 GWAS sites were associated to variables measured in Exp2-DRS. Only 2 GWAS sites, namely q-13 and q-46 were detected in both experiments (Figure 3), indicating stability across contrasting conditions (direct seeding and transplanting), although no gene with known relevant function to the trait reported in these regions (Table 4).

**Figure 4:**
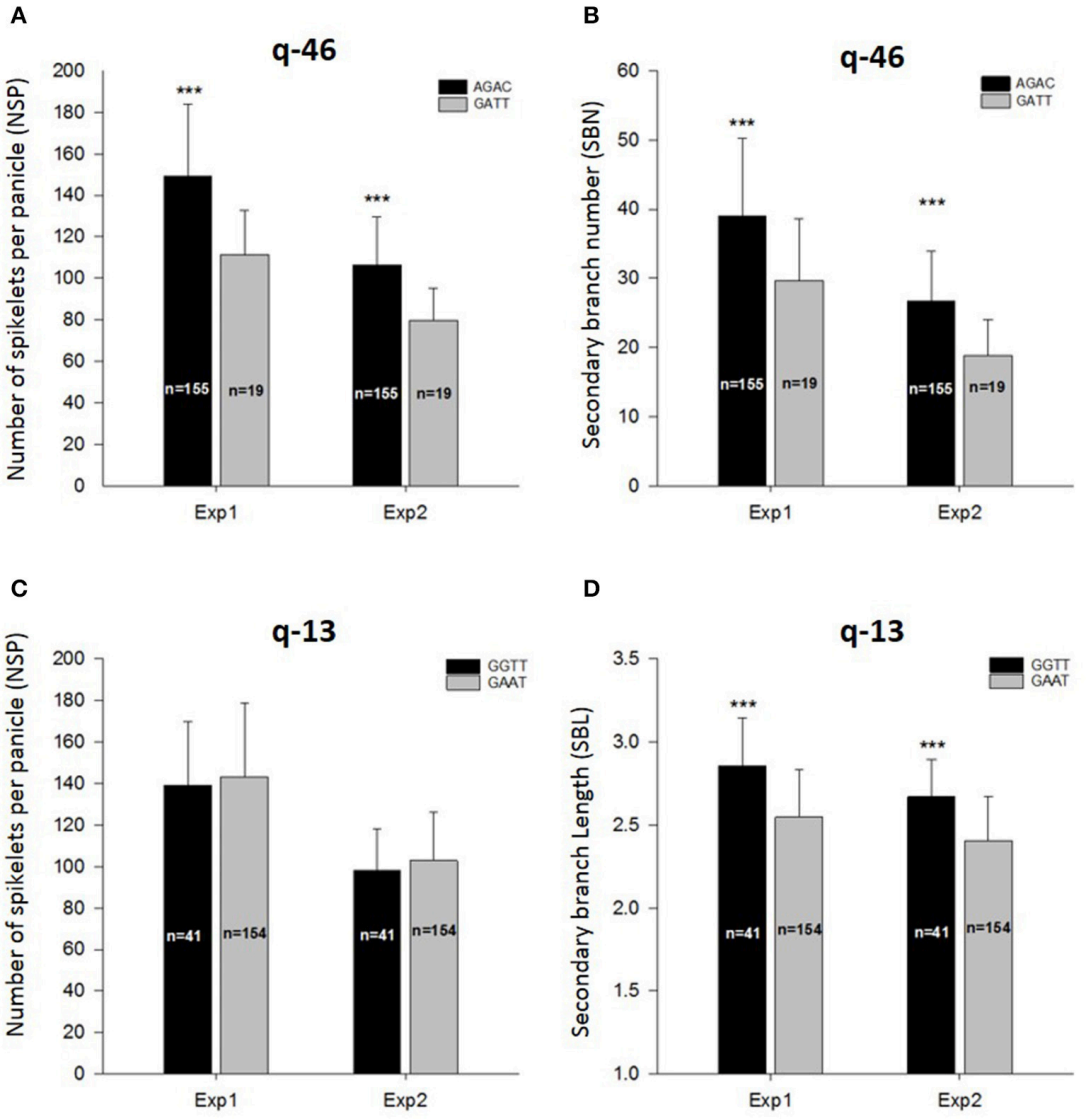
**Allelic combination for q-46 (A,B) and q-13 (C,D), and their effect on the number of spikelets per panicle, secondary branch number and secondary branch length**. ^***^Indicate significant differences at *p* < 0.001 between allelic combination for each experiment.

Within the LD block of site q-46 in chromosome 11 associated with SBN in Exp2-DRS and NSP in Exp1-TR, we detected four SNPs at positions 23,012,580, 23,012,600, 23,047,814, and 23,064,262 bp having significant associations with both trait and both environments. Within the indica panel, 155 accessions carrying the combination of AGAC at these markers showed superior values for NSP and SBN in both experiments (Figures 4A,B).

For site q-13 in chromosome 3 associated with SBLin Exp2-DRS and SBL in Exp1-TR, four markers at the positions 519,551, 519,745, 519,765, and 550,877 bp were found, whereby 41 accessions carrying the combination of GGTT had significantly superior values for SBL in both experiments (Figure 4D). However, these 41 accessions did not show higher values of NSP either in Exp1-TR or in Exp2-DRS (Figure 4C).

## Discussion

Despite some existing knowledge on the genetic control of the number of spikelets per panicle, there is little evidence on the added value for breeding of secondary traits, such as panicle architecture traits. This may be explained by the lack of undestanding of (i) the role of each panicle architecture component trait and their mutual compensations, (ii) the genetic architecture of panicle component traits, and (iii) the stability of the panicle component traits across contrasting environmental conditions.

The present study combined image analysis, GWAS and field trials under contrasting management conditions (transplanting vs. direct seeding) for the phenotypic and genetic dissection of the number of spikelets per panicle in rice. It was expected that the phenotyping of panicle architecture traits would provide further insight into trait-trait correlations and trade-offs defining the total number of spikelets per panicle, while the analysis of two contrasting conditions would provide insights into environmental and genetic control.

### Image analysis of panicle architecture enabled phenotypic dissection of number of spikelets per panicle

New open access image analysis tools such as the one used in this study (P-TRAP: AL-Tam et al., 2013) allowed us to evaluate the phenotypic contribution of panicle architecture to the number of spikelets per panicle in a large diversity panel. We observed a large range of variation and high heritability (Table 2) for the panicle architecture traits and the number of spikelets per panicle, suggesting a good performance of P-TRAP as a phenotyping tool. Crowell et al. (2014) using a different image analysis tool to dissect panicle architecture, also showed the advantage of image analysis over manual measurements to phenotype diversity panels.

Previous studies addressing panicle architecture highlighted the importance of each trait/gene according to its effect on the number of spikelets per panicle (Ikeda et al., 2004; Xu et al., 2004; Ando et al., 2008; Qiao et al., 2011; Hashida et al., 2013). In this study, NSP was significantly correlated with panicle architecture variables, confirming the positive effect of the number of branches (PBN, SBN) (Li et al., 2003; Wang et al., 2009) and branch length (PBL) (Xu et al., 2004; Li et al., 2009).

However, unlike in the aforementioned studies P-TRAP introduced new variables such as the number (SBN) and length (SBL) of secondary branches. Previous studies reported a positive correlation between SBN and NSP (Li et al., 2003; Wang et al., 2009). Hashida et al. (2013) observed an increased number of spikelets per panicle and secondary branches in an indica × japonica cross carrying the gene *OsSPS1*. Furthermore, several authors confirmed the importance of SBN over PBN as contributors to NSP in rice (Xu et al., 2004; Mei et al., 2006; Terao et al., 2010). To our knowledge studies addressing SBL are rare, mainly because of the difficulty to measure it manually and only Piao et al. (2009) showed that both SBL and SBN together contribute positively to the NSP. However, in this study, we did not observe a significant correlation between NSP and SBL (Table 3), in any of the growing conditions.

Variables related to the number of branches (PBN, SBN) were not significantly correlated with variables related to the length of branches (PBL), suggesting two independent ways to increase the NSP, either with a high number of branches or with long branches, involving trade-offs between them. The number of spikelets per panicle can be increased; either by increasing the number of branches (Xu et al., 2004; Wang et al., 2009; Qi et al., 2011) or by increasing the length of branches (Li et al., 2009). In fact, none of the GWAS sites associated with variables related to number and length of branches collocated, suggesting that the number and length of primary branches have an independent genetic control. To our knowledge, no study has addressed both length and number of branches to increase the number of spikelets per panicle. This may open a new way to increase NSP as suggested by Ando et al. (2008).

PL was significantly and positive correlated with NSP in Exp2-DRS and no correlation was found between PL and NSP in Exp1-TR. However, all panicle component traits (SBN, SBL, PBN, and PBL) were significantly and positively correlated with PL under both environmental conditions. This suggest that PL is not a simple trait and can be affected in a given genotype, by several component traits (SBN, SBL, PBN, or/and PBL) that are physiologically interacting and may involve environment dependent trade-offs. Thus, the low correlation observed between PL and NSP in this study can be explained by the effect of the growing conditions on PL and/or the different ways a genotype can achieve a large number of spikelets per panicle (either with long panicles or with short but dense panicles).

### GWAS on panicle architecture dissects genetic control of number of spikelets per panicle

The exploration of a diversity panel using GWAS and image analysis of panicle architecture allowed us to find 25 new loci that were involved in the genotypic variation of NSP a complex trait (Table 3). A total of 23 GWAS sites had significant associations (*p* < 1.00 E^−4^) with panicle architecture traits. Furthermore, SNPs with high association probabilities were more likely to be close to previously identified candidate genes, e.g., *Osjag* in q-1 and *OsCKX2* in q-3 with p values of 7.70E−5 and 1.54E−5 respectively. Besides, image analysis allowed us to measure new variables, such as SBN, that were related with the number of spikelets per panicle and associated with new GWAS sites, some of which related with known genes (*OsCKX2, OsMADS5*). Thus, this GWAS study identified many true genotype-phenotype associations, and detected GWAS sites that were associated to already known genes with a highly significant effect on the number of spikelets per panicle (Table 4).

Ten GWAS sites associated only with NSP remain interesting markers that might be associated with a component trait of NSP that was not measured in this study (Qiao et al., 2011; Hashida et al., 2013).

Numerous markers with significant but small effect were also found. This was also the case in other association studies for panicle architecture traits in rice (Crowell et al., 2016) and sorghum (Morris et al., 2013; Hmon et al., 2014), confirming that panicle architecture is controlled by many QTLs with small effect. So far, several GWAS sites having significant associations at *P* < 5.5 E^−4^ and associated with genes controlling panicle architecture have been identified and characterized independently. The genes such as *MOC1, LAX1/2, OsCKX2, SP1, DEP1/2/3*, and *IPA1/WFP* were found to modify panicle architecture and were suggested as candidates to improve the number of spikelets per panicle in rice through molecular breeding (Komatsu K. et al., 2003; Ashikari et al., 2005; Miura et al., 2010; Qiao et al., 2011). Interestingly, Wang and Li (2011) and Liang et al. (2014) suggested that their effect on the final number of spikelets per panicle would be greater if they were all included in the breeding design, as they all act in an additive way.

Co-localization of genetic control of various traits in the same genomic region was rare in this study (7 out of 50 GWAS sites) compared with other studies (Rebolledo et al., 2015). Considering the LD region of 75 kb, we found two co-localizations between panicle architecture traits and NSP (q-9, q-43) suggesting that component traits derived from the image analysis of panicle architecture strengthened the analysis of NSP.

Globally, in our study only 14 GWAS sites out of 50 were significantly associated with NSP only. Four out of the 14 GWAS sites for NSP were related to known, cloned genes; two GWAS sites were associated with genes with a function affecting panicle density (q-22, gene *GSK22*) or floral organ identity (q-25, gene *NL1*) and two genes with a function on tillering ability (q-23, *OsHRZ2* gene) or carbon and nitrogen content (q-6, gene *OSAAT7*).

### Contrasting field trials revealed specific and common GWAS sites

The Exp2-DRS showed lower values for NSP, PBN, SBN than Exp1-TR across the 225 varieties (Table 2). The negative effect of direct seeding on the number of spikelets per panicle was reported by Huang et al. (2011) for super hybrid rice, showing that a smaller amount of nutrients available under direct seeding at panicle initiation affected panicle branching and the final number of spikelets per panicle. This result reflects the high degree of sensitivity to the environment of panicle architecture, making the detection of genetic controls more difficult.

However, we detected two GWAS sites consistently in both transplanting and direct seeding conditions. Site q-13 was stable across conditions and was associated with branching pattern (SBL) but did not have a significant effect on NSP. This result supports the idea that the length of the branches has a smaller effect on the number of spikelets per panicle (Ando et al., 2008).

Site q-46 was also stable in Exp1-TR and Exp2-DRS and controlled both SBN and NSP. Thus, new variables measured in this study using image analysis led to the discovery of new genetic regions with a significant effect on NSP across two different rice growing conditions.

Hence, despite the strong environment effect, some genomic regions associated with multiple traits point to the potential for improvement of yield potential through increased number of spikelets per panicle for both transplanting and direct seeding conditions. But overall, it appears that molecular breeding for increased NSP can build on more relevant loci when targeting a specific environmental and cultural practice.

## Author contributions

MR designed the project, MR and AP analyzed and interpreted the data, also they wrote the manuscript. MD led the GRiSP Global Rice Phenotyping Network which this study was part of. All authors made important intellectual contributions. JD and DC performed the bioinformatic analysis of the GBS data.

### Conflict of interest statement

The authors declare that the research was conducted in the absence of any commercial or financial relationships that could be construed as a potential conflict of interest.
